# A study on the global patterns in the design and development of ventricular assist devices: a visualization approach

**DOI:** 10.3389/fcvm.2025.1371443

**Published:** 2025-01-31

**Authors:** Ajay K. Sood, A. K. Prasada Rao

**Affiliations:** ^1^ School of Engineering and Technology, BML Munjal University, Gurugram, India; ^2^ Department of Mechanical Engineering, GITAM Institute of Science and Technology, Gitam Deemed to be University, Visakhapatnam, Andhra Pradesh, India

**Keywords:** ventricular assist device, LVAD, blood pump design, computational fluid dynamics, continuous-flow pump

## Abstract

**Introduction:**

Ventricular assist devices (VADs) are lifesavers for people with advanced heart failure. The design of these devices has undergone drastic changes over time with the latest designs being far more efficient, small, lightweight, and more user-friendly. This study aims to analyze publications using bibliometric analysis and see the progress and identify key themes, trends, and collaboration networks.

**Method:**

Data relevant to this study were obtained from Scopus and Web of Science databases from 1990 to 2023. Data analysis was done using Biblioshiny which is an R-based software and is part of RStudio and Microsoft Excel to analyze collaboration between countries, authors, keyword analysis, trend topics, and evolution of various themes related to this study.

**Results:**

A total of 489 published documents were analyzed, and these documents were from 158 different sources and 1,753 authors. The top contributing journals were *Artificial Organs* and *Asaio Journal* with 116 and 81 publications, respectively. The top contributing authors in terms of total documents were Nose Y (35) and Throckmorton A (30) and in terms of total citations were Pagani F (2005) and Mehra M (1952). Top countries include the USA, China, and Germany. The trend topics include miniaturization, machine learning, wireless, shear flow, and fiber-optic sensors.

**Discussion:**

The latest technological advancements in VAD design are making them a more suitable choice for a large number of patients. This bibliometric work will aid in identifying the newest trends and developments in this field and highlight the areas where more research is needed. These data are crucial for driving innovation in this field and for improving the lives of patients who depend on VADs. Future studies can be conducted to explore the use of artificial intelligence and machine learning that can learn from data about patients and then adapt as per the requirements of the patients.

## 1 Introduction

Ventricular assist devices (VADs) are real lifesavers and provide a lifeline to many patients who are medically at high risk for transplant by providing them with long-term support. Broadly, VADs can be classified into two major types: left ventricular assist devices (LVADs) (1, 2) and right ventricular assist devices (RVADs) (3–5). LVADs provide a long-term solution as they can be used as destination therapy (DT) for patients. The implantation of a ventricular assist device (VAD) involves open-heart surgery (6, 7). The main focus of the present work is to study how durable LVAD designs have evolved across the globe from 1990 to 2023. Today's LVADs are smaller and lighter than earlier designs, but they still carry certain risks, including bleeding, infection, and the formation of blood clots. Therefore, researchers across the globe are working to optimize various design parameters of LVADs and improve their overall efficiency. This can lead to increased comfort for patients needing it for long-term support.

Bibliometric analysis has not been used to date to study the growth in the domain of design and development of VADs. To address this gap, the authors used bibliometric analysis to comprehensively study the important research trends in this field. Bibliometric analysis is a quantitative and qualitative approach used for studying literature in a specific field using statistical and mathematical methods. This approach helps recognize the trends in a research area and the most prominent collaboration network between various authors, institutions, and countries (8–12). Bibliometric analysis, in contrast to systematic reviews (13, 14) that aim to provide answers to specific research queries based on a limited number of publications, provides comprehensive information related to the literature in a particular field. Similarly, bibliometric analysis differs from scoping reviews (15, 16), which focus on identifying the nature and scope of research evidence.

Improvements in VAD design, surgical techniques, and aftercare have made these devices a crucial treatment option for a challenging group of patients with heart failure (17). Continuous-flow VADs, the type of VAD most commonly used today, can now last for approximately 5 years on average and even more than 10 years in some exceptional cases (18). Over the past decade, these devices have become a primary treatment option for patients who have been on the heart transplant waiting list for a long time and for those who are not suitable for transplantation (19–21). Drawing on information about the evolution of themes with time and the global collaboration network between authors, countries, and institutes, this paper aims to elucidate the research trends in the design and development of ventricular assist devices (VADs). The findings will provide valuable references for future research. Furthermore, by gaining insights into the research trend topics in this domain, policymakers and funding agencies can make informed decisions about where to allocate research resources and prioritize future research efforts.

## 2 Methodology

**Figure 1:**
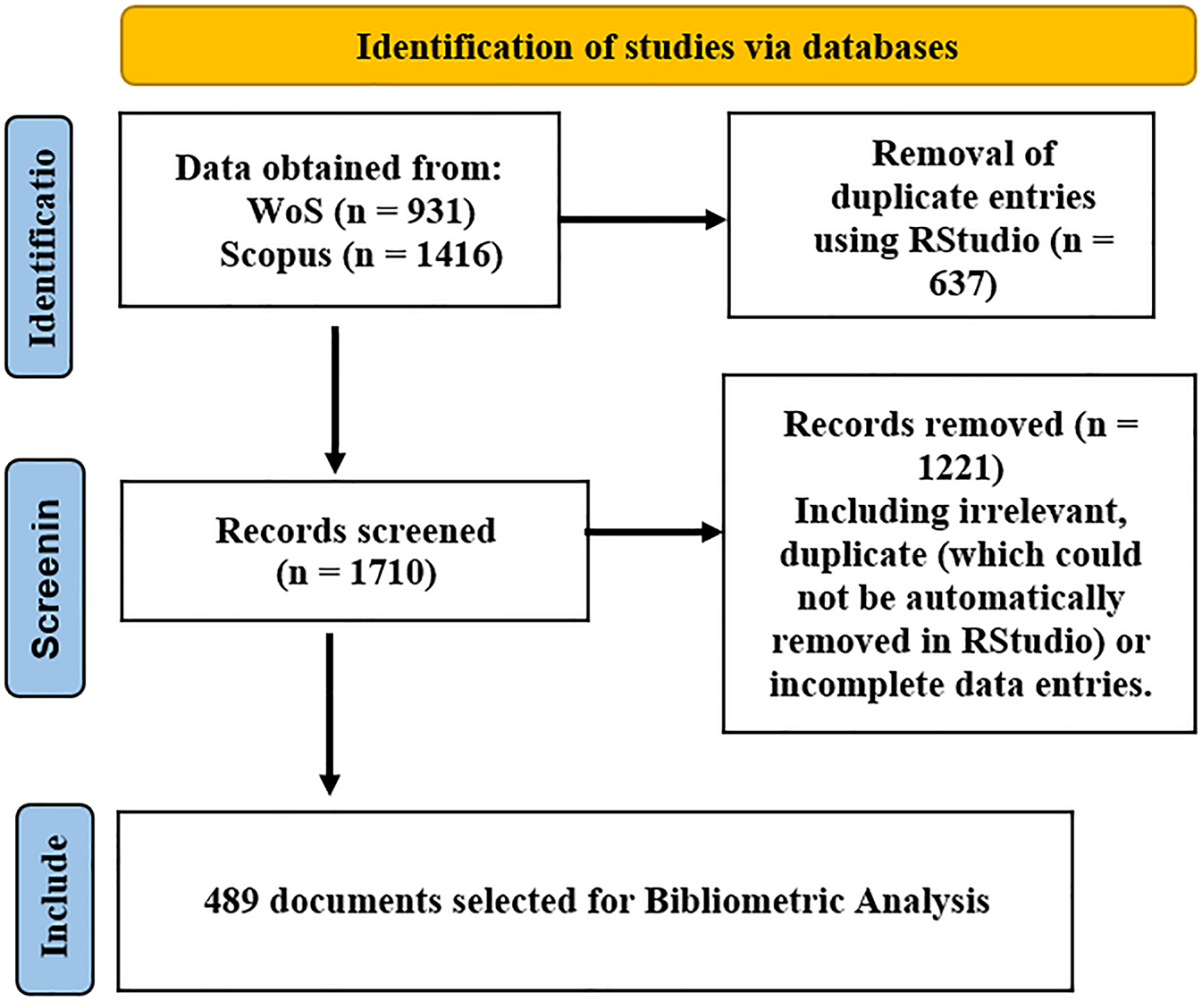
Flowchart used for data selection process based on PRISMA.

The steps involved in the bibliometric analysis (refer to Figure 1) include collecting data from relevant sources, cleaning the data to remove irrelevant and duplicate entries (22), and performing the analysis using a bibliometric tool called Biblioshiny (23) (which is based on R and is a part of RStudio). The final step involves plotting and explaining various network plots, thematic maps, and bar charts (see the progress and trend topics in this domain).

### 2.1 Data collection

For this study, data was sourced from two different databases, namely, Elsevier's database Scopus (https://www.scopus.com) and Web of Science (WoS) (https://www.webofscience.com; from Clarivate Analytics). Data was collected from 1990 to 2023. Both Scopus and WoS are well-known databases for collecting data required for bibliometric analysis. A total of 1,416 papers from Scopus and 931 documents from WoS were downloaded using the keywords ventricular assist device, VAD, axial flow, centrifugal flow, continuous-flow, design, model, computational fluid dynamics (CFD), simulation, and computational fluid with a focus on getting papers related to VAD research, either experimentally or computationally, for design, development, or fabrication purposes using the following search string:

TITLE-ABS-KEY ((“ventricular assist device” OR vad) AND (“axial flow” OR “centrifugal flow” OR “continuous flow” OR “axial pump” OR “centrifugal pump” OR “continuous pump”) AND (design OR model OR cfd OR simulation OR “computational fluid”)) AND (LIMIT-TO (LANGUAGE, “English”))

### 2.2 Data merging and cleaning

For merging data from Scopus and WoS databases, RStudio was used. With the help of a code, the two databases were merged (24) and duplicate entries were removed, with a total of 637 duplicate entries removed. After merging and cleaning in RStudio, the data file was exported to Microsoft Excel. In Microsoft Excel, manual cleaning of data was performed to remove entries with missing data, i.e., author names, and some duplicate entries. Moreover, some irrelevant papers that did not deal with the design and development aspects of VAD were removed. The final cleaned data set consisted of a total of 489 documents of various kinds (articles, conference papers, review papers, books, and book chapters).

### 2.3 Data analysis

The bibliometric analysis was performed on the cleaned dataset using the R-based Biblioshiny software, and various network plots such as country collaboration network, institute collaboration network, and author collaboration network were made. There are multiple software choices available for conducting bibliometric analysis (if data is from a single source) such as CiteSpace, Gephi, Bib Excel, and VOSviewer). However, there is only one good option (Biblioshiny) available for constructing network plots from merged data obtained from multiple databases, such as Scopus and WoS. Biblioshiny is an open-source statistical tool with a good graphical user interface (25). Microsoft Excel was also used for plotting pie and bar charts. A correlation analysis was conducted to find the correlation between the number of annual citations and the age of the documents (based on the year of publication).

## 3 Results

This section presents the results of this study. Different analyses have been performed to determine the progress in this research domain.

### 3.1 Data overview

The data consists of 489 publications from 158 different sources (journals, books, etc.) published by 1,753 authors (number of authors of single-authored documents = 15 and number of single-authored documents = 19 and international co-authorships = 10.02%).

### 3.2 Analysis of published documents

Articles (*N* = 339) account for 69.33% of the total publication collection, and conference papers (*N* = 100), review papers (*N* = 35), book chapters (*N* = 8), and books (*N* = 7) account for 20.45%, 7.16%, 1.63%, and 1.43%, respectively. The average number of citations per document is 25.91. The annual growth rate of the documents is 2.06%. The average number of co-authors per document is 6.45. The first publication in this domain of research is from 1990 (26) where authors investigated the use of the first Hemopump (a temporary VAD) on humans.

**Figure 2:**
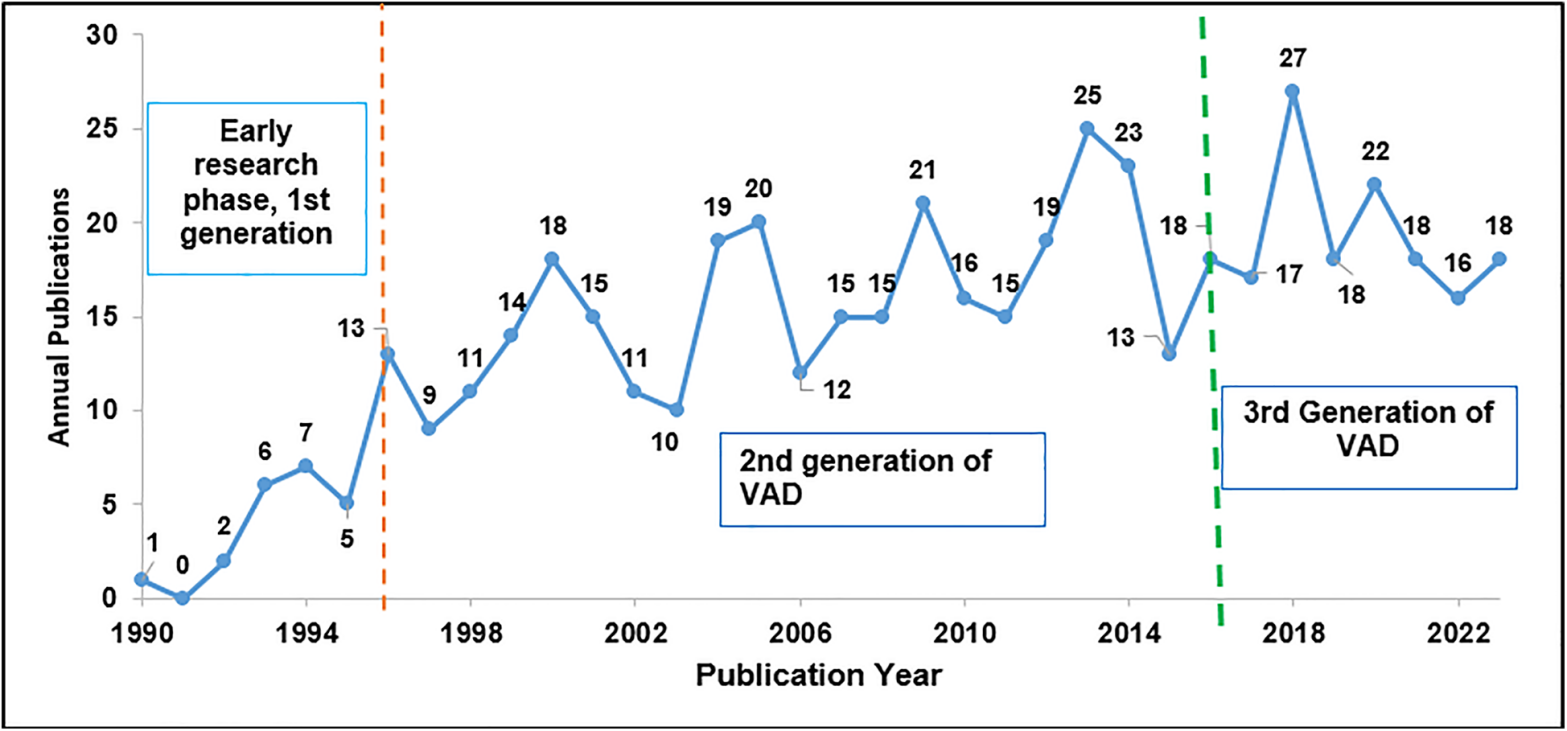
Total annual publications (1990–2023).

Figure 2 shows publications from 1990 to 2023. This study considered data from 1990 onwards. The early research phase (first phase) started from the 1950s to the late 1990s, during which the first VAD was used in humans in 1963 (27). The second phase of the research related to the design and development of VADs can be considered to be from 1998 when second-generation VADs were tested on humans for the first time (28). Significant work in the design and development of second-generation VADs was conducted by various scholars (29–34). These published works provide a thorough summary related to the design and development of various second-generation VADs, such as HeartMate II, HeartWare VAD, and Medtronic HVAD, and the challenges faced.

Second-generation VADs were smaller, more durable, and more reliable than first-generation devices but were still associated with several complications, including stroke, infection, and pump thrombosis. The maximum number of publications is 310 (63.39%) which are from the time domain of using second-generation VADs. The third phase in the design and development of VADs can be considered from 2017 to date. The third-generation VADs were used for the first time in 2017. They were named HeartMate 3 LVADs at the University of Michigan (35). These are the latest series of VADs used in humans since 2017. A lot of research is still ongoing for further improvement in its design and performance (36–39).

Annual publications reached double figures for the first time in 1996, and since then, the number of publications has consistently increased compared to previous years. The number of documents published was 43 (8.80%) from 1990 to 1997, 310 (63.39%) from 1998 to 2016, and 136 (27.81%) from 2017 to 2023, which shows a clear upward trend that more research has been conducted in this domain during these periods.

The total citations of articles was 11,078 (77.60%), followed by conference papers, review papers, books, and book chapters with citations equal to 1,808 (14.27%), 1,002 (7.91%), 16 (0.13%), and 12(0.09%), respectively. This clearly shows that article papers are the most significant type of publication.

### 3.3 Analysis of authors and their collaboration network

The top 10 authors based on their total publications are shown in Table 1. The author affiliations shown in the table are obtained from their most recent publication in the data collected. Other information includes total citations, h-index value, and the years the authors published their first and latest papers. All of the 10 top authors are affiliated with institutions in the USA, which shows that most productive research work is being conducted or has been conducted in the USA. Nose Y (*N* = 35) and Throckmorton A (*N* = 35) have the highest number of publications, while Oslen D has the highest h-index of 17. Notably, Throckmorton A, Antaki J, and Frazier O are the only authors who have remained actively involved in research publications related to this field in the last 5 years.

**Table 1:** Top 10 most productive authors (based on total publications).

Author name	Affiliation	No. of papers	Total citations	h-index	Year of first publication	Year of last publication
Nose Y	Michael E. DeBakey, Department of Surgery, Baylor College of Medicine, Houston, TX, USA	35	646	14	1993	2013
Throckmorton A	BioCirc Research Laboratory, School of Biomedical Engineering, Science, and Health Systems, Drexel University, Philadelphia, Pennsylvania, USA	30	544	14	2003	2023
Allaire P	University of Virginia | UVa · ROMAC Laboratories, USA	29	687	16	1996	2015
Olsen D	Utah Artificial Heart Institute, Salt Lake City, UT, USA	27	725	17	1992	2010
Frazier O	Innovative Design and Engineering Applications Laboratory, The Texas Heart Institute, Houston, Texas, USA	23	1412	15	1,990	2023
Wood H	Professor of Mechanical and Aerospace Engineering, University of Virginia, USA	22	553	15	1999	2013
Glueck J	Albert Einstein College of Medicine, Bronx, NY, USA	20	292	9	1994	2013
Antaki J	Department of Biomedical Engineering, Carnegie Mellon University, Pittsburgh, PA, USA	18	547	14	1993	2020
Benkowski R	MicroMed Technology, Inc., Houston, Texas, USA	16	461	11	1995	2010
Noon G	Department of Surgery, Baylor College of Medicine, Houston, Texas, USA	12	645	12	1993	2001

**Figure 3:**
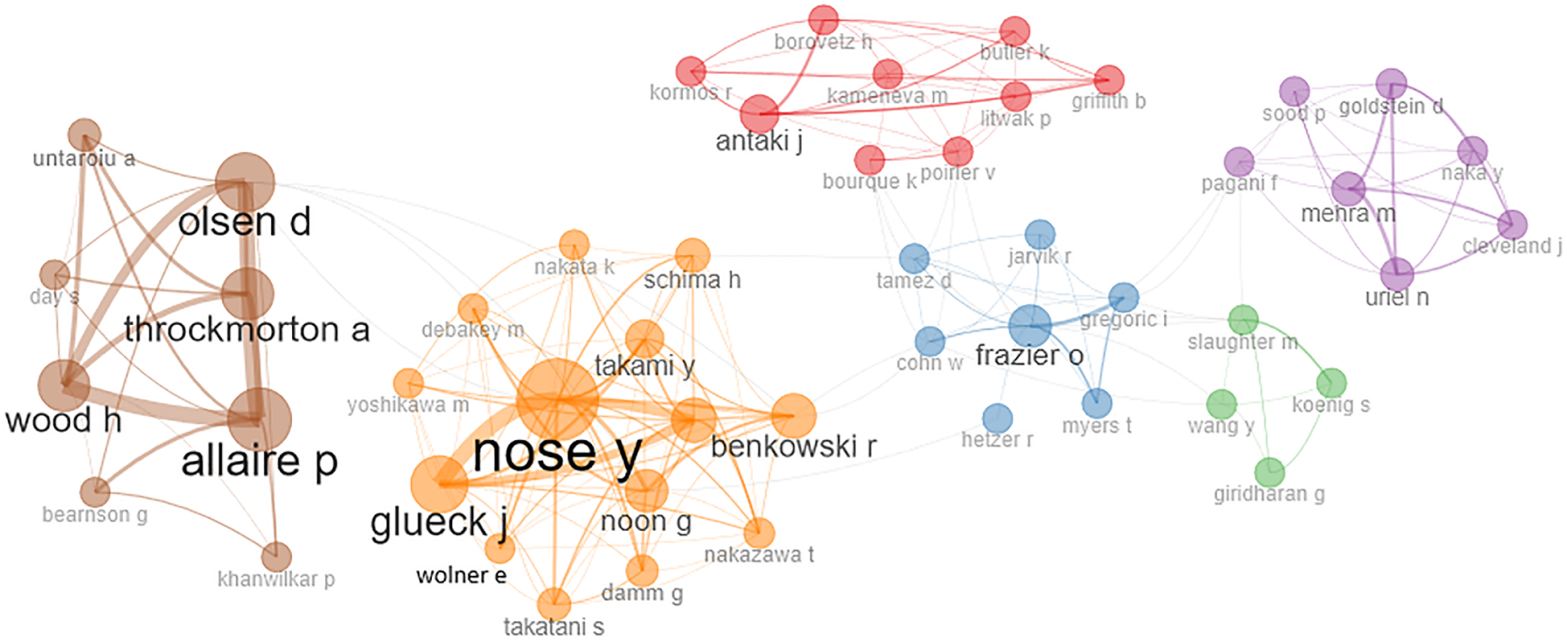
Collaboration network of top 50 authors.

The collaboration network between the top 50 contributing authors is shown in Figure 3. In this network, the nodes represent the authors, the size of the nodes indicates the number of publications, and the lines joining these nodes represent the collaboration strength. Six different clusters can be seen in six different colors. Authors with strong collaboration can be seen in the same cluster. All of the top 10 authors shown in Table 1 can be seen in various clusters indicating that these authors have strong collaboration networks. The largest cluster (orange) consists of 14 authors, 10 from the USA and 2 each from Austria and Japan. All authors from the second (red) cluster (nine authors), third (brown) cluster (eight authors), fourth (purple) cluster (seven authors), fifth (blue) cluster (seven authors), and sixth (green) cluster (five authors) are from the USA. This suggests that the majority of significant work in this field has been conducted in the USA, as most of the top authors are from there. Consequently, it indicates that high-quality research work is being conducted in the USA.

### 3.4 Identifying the structure of scientific knowledge using bibliographic coupling of sources

**Figure 4:**
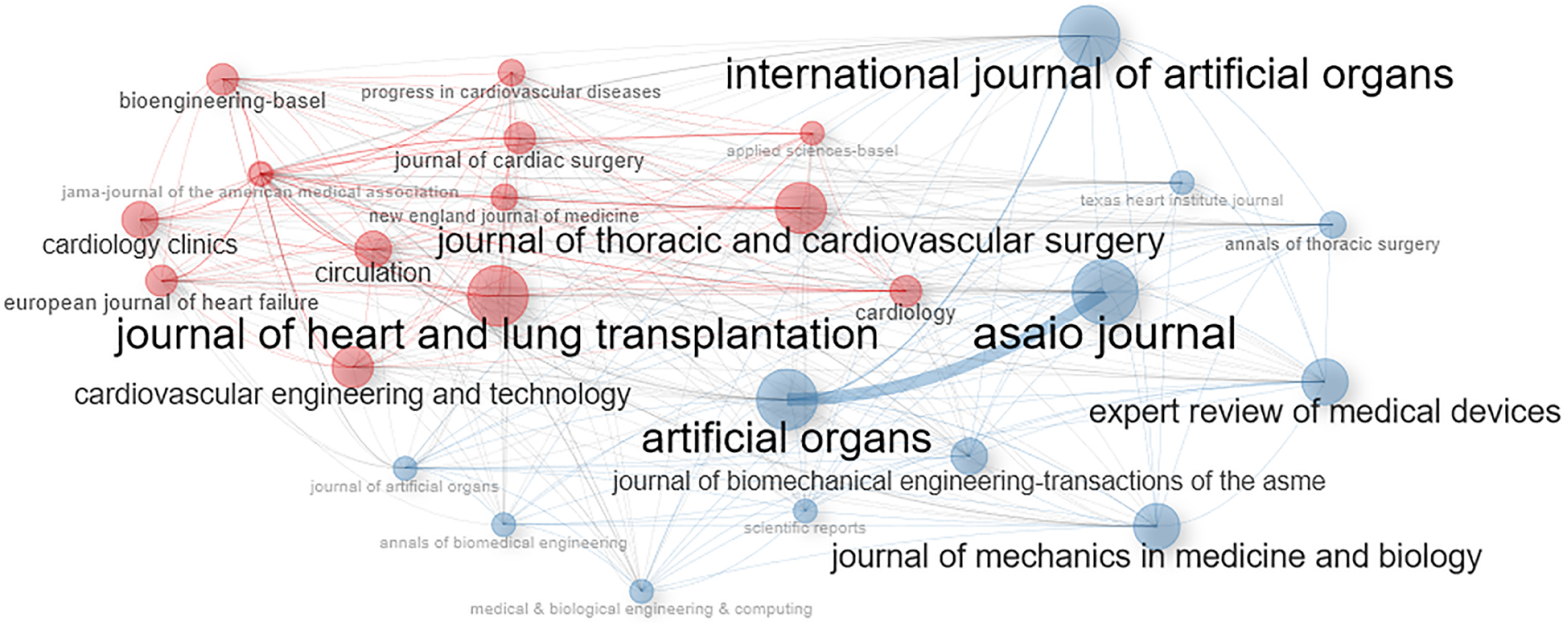
Bibliographic coupling of sources.

Figure 4 shows clustering by coupling which is a useful method for identifying the structure of scientific knowledge. This method helps researchers understand the latest advances and trends in a particular research domain. The clusters in Figure 4 represent the sources of publications; we grouped sources where various authors have published work on similar themes. The biggest cluster (red) consists of 18 different sources that have published papers on the same theme (experience related to the use of VAD). The second cluster (blue) consists of three sources, with a common theme related to heart transplantation and heart failure.

### 3.5 Analysis of the most productive countries and their collaboration network

The top 10 most productive countries in terms of number of publications are shown in Table 2. Their total citations are also shown in the same table. The USA is the most productive country in terms of number of publications (*N* = 282) which accounts for 57.66% of the total publications. China ranked second with a total of 33 publications, and Germany ranked third with 29 publications. Japan and the UK ranked fourth and fifth with 26 and 13 publications, respectively. The top countries were identified based on the corresponding authors for the publications. In terms of citations, only five countries had more than 100 citations with the USA ranking first with 8,937 citations, Germany ranking second with 899 citations, and the Czech Republic ranking third with 373 citations. Austria and The Netherlands ranked fourth and fifth with 371 and 311 citations, respectively. From the country production, it is obvious that most of the research related to the design and development of VADs was conducted in the USA.

**Table 2:** Top 10 most prominent countries based on number of publications.

S. no	Country	Country production	% of total production	No. of citations	% citations
1	USA	282	57.66	8937	71.64
2	China	33	6.74	119	0.95
3	Germany	29	5.93	899	7.20
4	Japan	26	5.31	309	2.47
5	UK	13	2.65	171	1.37
6	Singapore	11	2.24	143	1.15
7	Italy	9	1.84	112	0.89
8	Austria	7	1.43	371	2.97
9	Brazil	7	1.43	65	0.52
10	Canada	7	1.43	118	0.94

**Figure 5:**
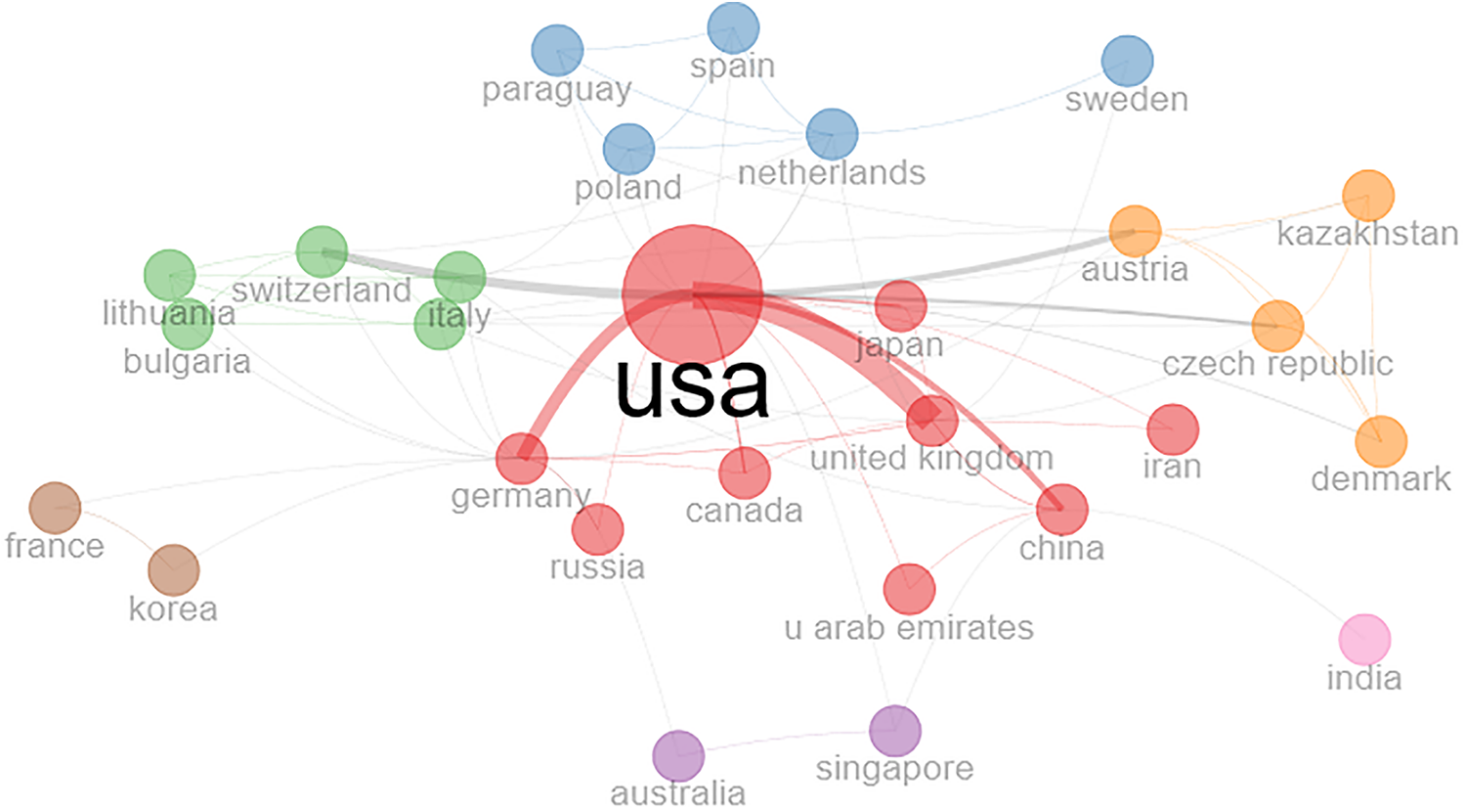
Country collaboration network of partner countries.

The country collaboration network of 28 countries, out of 38 countries that have published papers in this domain, has been presented in Figure 5. Seven clusters can be seen in this figure, out of which the red cluster is the largest with nine countries (i.e., USA, Germany, Japan, UK, Canada, China, UAE, Iran, and Russia). All the top 10 most productive countries as shown in Table 2 can be seen in this figure also, which shows that all of the top 10 countries have good global research collaboration networks.

### 3.6 Analysis of the most active journals

The journals with the number of papers in the domain of VAD design and development are shown in Table 3. Out of the 105 different sources included in the present work, 10 journals had the highest number of publications, as shown in Table 3. *Artificial Organs* ranked first with 116 publications, while *Asaio Journal* ranked second with 81 publications. The top 10 journals include 283 publications which account for 57.87% of the total publications (*N* = 489) and a total of 7,579 citations which account for 59.80% of total citations (*N* = 12,672).

**Table 3:** Top 10 journals with the highest number of publications.

Rank	Journals	Publishing house, country	No. of publications	No. of citations	Scopus quartile	WOS
1	*Artificial Organs*	Wiley Online Library, UK	116	2,455	Q2	SCIE
2	*Asaio Journal*	Lippincott Williams & Wilkins, USA	81	1,746	Q1	SCIE
3	*Journal of Heart and Lung Transplantation*	Elsevier Science Inc., USA	21	1,384	Q1	SCIE
4	*International Journal of Artificial Organs*	Sage Publications Ltd., England	16	208	Q3	SCIE
5	*Annals of Thoracic Surgery*	Elsevier Science Inc., USA	14	744	Q1	SCIE
6	*Texas Heart Institute Journal*	Texas Heart Institute, USA	9	473	Q3	SCIE
7	*Journal of Thoracic and Cardiovascular Surgery*	Mosby-Elsevier, USA	8	174	Q1	SCIE
8	*Journal of Artificial Organs*	Lippincott Williams & Wilkins, USA	7	88	Q3	SCIE
9	*European Journal of Cardio-Thoracic Surgery*	Oxford University Press Inc., USA	6	245	Q1	SCIE
10	*Journal of Cardiac Surgery*	Wiley Online Library, UK	5	62	Q2	SCIE

### 3.7 Analysis of the most cited publications

Table 4 shows the top 10 most cited publications. Normalized total citation is used to measure the citation impact of a research paper when compared to other papers published in the same research domain (40–42).

**Table 4:** Top 10 most cited publications.

S. no	Paper (first author, year, journal)	DOI	Total citations	Average citations per year	Normalized total citations
1	Mehra MR, 2019, *New Engl J Med*	10.1056/NEJMoa1900486	711	118.50	11.60
2	Mehra MR, 2017, *New Engl J Med*	10.1056/NEJMoa1610426	599	74.88	7.67
3	Rogers JG, 2017, *New Engl J Med*	10.1056/NEJMoa1602954	525	65.63	6.73
4	Moazami N, 2013, *J Heart Lung Transpl*	10.1016/j.healun.2012.10.001	220	18.33	9.14
5	Larose JA, 2010, *ASAIO J*	10.1097/MAT.0b013e3181dfbab5	197	13.13	5.39
6	Netuka I, 2015, *J Am Coll Cardiol*	10.1016/j.jacc.2015.09.083	180	18.00	6.00
7	Soliman OII, 2018, *Circulation*	10.1161/CIRCULATIONAHA.117.030543	170	24.29	7.45
8	Griffith BP, 2001, *Ann Thorac Surg*	10.1016/S0003-4975(00)02639-4	163	6.79	3.53
9	Noon GP, 2001, *Ann Thorac Surg*	10.1016/S0003-4975(00)02634-5	153	6.38	3.32
10	Kamdar F, 2009, *J Heart Lung Transplant*	10.1016/j.healun.2009.01.005	136	8.50	4.29

The first paper in Table 4 is a comparative study between the performance of centrifugal and axial flow types of LVADs which suggests that the use of the centrifugal type is better in preventing pump thrombosis (43). The second paper discusses the magnetically levitated centrifugal pump and its role in reducing pump thrombosis while using LVADs (44). The third paper is a comparative study on the use of centrifugal and axial flow devices for patients who did not meet the eligibility criteria for transplant (45). The fourth paper discusses the comparison of axial and centrifugal pumps and bearing design in terms of their reliability and biocompatibility (46). The fifth article discusses the design aspects related to the left ventricular assist device (LVAD) HeartMate II in terms of its evolution and use (47). The sixth paper deals with the design aspects and performance of the HeartWare VSD device which consists of a centrifugal-type pump (48). The seventh paper focuses on deriving and validating a new failure model using continuous-flow LVADs (49). The eighth publication focuses on HeartMate II development and its first clinical use (50). The ninth publication discusses the use and performance of the MicroMed DeBakey VAD which is an axial flow type of VAD (51). The 10th paper discusses the results and effects of the use of centrifugal, axial, and pulsatile left VADs on heart failure patients (52).

### 3.8 Analysis of keyword co-occurrence network

**Figure 6:**
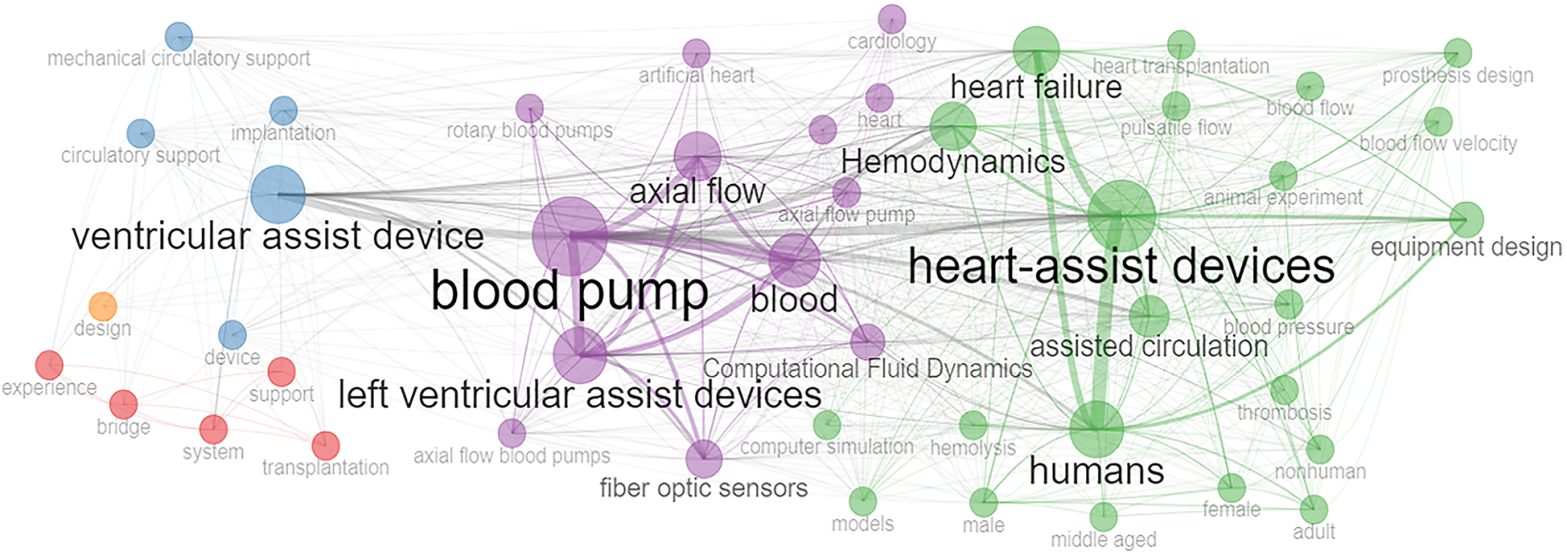
Keyword co-occurrence network.

The network related to the co-occurrence of keywords provides a graphical representation of the relationships between different keywords in publications, making it a very useful tool for highlighting important themes within a given research area (53). Each node in the network represents a keyword, with its size indicating the frequency of that keyword in the database. The thickness of the lines connecting the nodes shows the strength of the co-occurrence of keywords. Figure 6 shows five clusters in the keyword co-occurrence network. The first cluster (green) has 22 nodes, each representing a keyword. Some significant keywords in this cluster included assisted circulation, hemodynamics (54), computer simulation, blood flow velocity, equipment design (55), and thrombosis (56). This clearly shows research on the design, development, and use of VADs to prevent human heart failure. The second biggest cluster (purple) has 13 nodes with the most frequently used keywords being blood pump, blood, rotary flow blood pumps (57), left ventricular assist devices, and computational fluid dynamics (58, 59). This again shows research focused on the design, development, and use of VADs to pump blood. The third cluster (blue) has five nodes, each representing a keyword, such as ventricular assist device, mechanical circulatory support (60), implantation, and device.

### 3.9 Thematic analysis

#### 3.9.1 Thematic evolution (evolution of themes with time)

**Figure 7:**
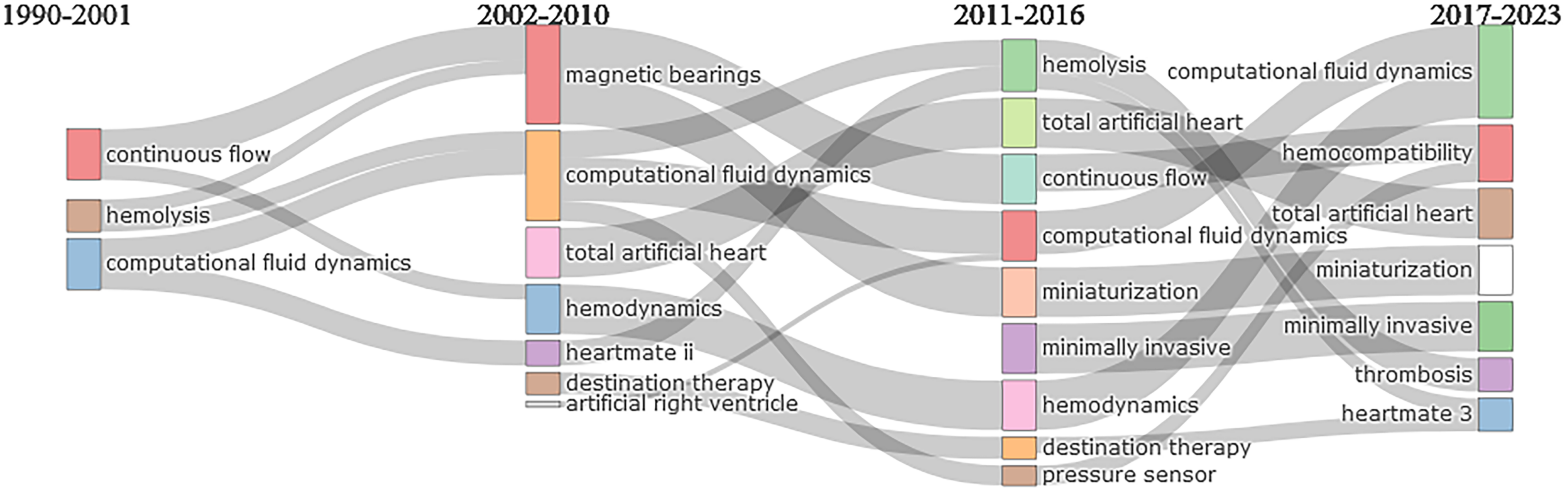
Thematic evolution.

The thematic evolution plot is a visual representation of how the main topics in a field of study change with time. It is based on a statistical analysis of the most common keywords in the domain of research. This analysis projects the keywords onto a simplified space, while keeping as much of the original information as possible. There are lines (Figure 7) connecting various keywords from different time zones which show the progress of research related to that keyword with time. The thick lines represent a strong correlation across different time zones. Figure 7 shows the thematic evolution of various topics related to VAD design and development with the passage of time. It can be divided into four time zones based on technological advancements.

• Early research in VAD design and development (1990–2001): Important milestones during this duration included the design and development of first-generation VADs such as early pulsatile pumps and HeartMate I and their limitations and challenges involved in their use such as blood damage, device safety, effectiveness, infection, and thrombosis. Other significant developments in this era included the REMATCH trial (published in 2001), bridge-to-transplantation (BTT), and the introduction of axial flow pumps in VADs. Figure 7 shows the important thematic terms during this domain, such as as hemolysis, continuous flow, and computational fluid dynamics (early use of this technique in a limited manner). Most common terms appearing in thematic analysis such as VAD, LVAD, centrifugal pump, axial flow pump, blood, humans, and design have not been shown in this figure so other significant terms can be highlighted.
• Technological advancements and commercialization (2002–2010): This time zone highlights the introduction of continuous-flow VADs, the use of axial flow devices, improved device durability, and reduction in patient complications in comparison to the previous time zone. Other significant developments in this era included conducting of HeartMate II trials and publication of the results of these trials. It showed a dominance of bridge-to-transplant, improved patient survival rates, and significance of continuous-flow devices. Figure 7 shows some significant keywords during this time zone which are summarized as follows:
  - *Themes with increased frequency*: computational fluid dynamics, magnetic bearings
  - *New/emerging themes*: HeartMate II, destination therapy, miniaturization
  - *Other significant themes*: hemolysis, hemodynamics, pump thrombosis, continuous-flow VAD
• Clinical expansion with advanced trials and device miniaturization (2011–2016): This time zone witnessed the increased use of continuous-flow VADs and miniaturized VADs such as HeartWare VAD and wearable devices and the adoption of less invasive implantation procedures. Other significant developments in this era included publication of results of the ADVANCE trial which evaluated the HeartWare VAD in patients with end-stage heart failure. These trials showed that small-sized and less invasive VADs were equally effective as the previously used continuous-flow devices, thus enabling the clinical use of VADs for children as well. Figure 7 shows some significant keywords during this time zone which are summarized as follows:
  - *Themes with increased frequency*: continuous flow, miniaturization, minimally invasive, computational fluid dynamics.
  - *Emerging themes*: children, fiber-optic sensors, pressure sensors, total artificial heart
  - *Other significant themes*: hemolysis, hemodynamics, portability
• Era of technological advancements leading to advanced device designs (2017–2023): This time zone has seen significant improvements in the VAD designs. This time zone witnessed significant improvements in LVAD design and development-related technology, including MagLev technology, better hemocompatibility, and the consolidation of destination therapy as a long-term solution. Additionally, there was a surge in comparative studies, expanded patient populations, and innovations in AI and remote care. The MOMENTUM 3 trial and publication of its results, which compared the performance of HeartMate 3 (a magnetically levitated, centrifugal flow LVAD) with that of the older HeartMate II (an axial flow device), is another important milestone during this time zone. Figure 7 shows some significant keywords during this time zone which are summarized as follows:
  - *Themes with increased frequency*: hemocompatibility, thrombosis, destination therapy, multicenter trials, computational fluid dynamics, minimally invasive, miniaturization
  - *Emerging themes*: machine learning, artificial intelligence, total artificial heart, wireless, HeartMate 3
  - *Other significant themes*: modeling, pediatric VAD, magnetically levitated, shear stress

#### 3.9.2 Thematic map

**Figure 8:**
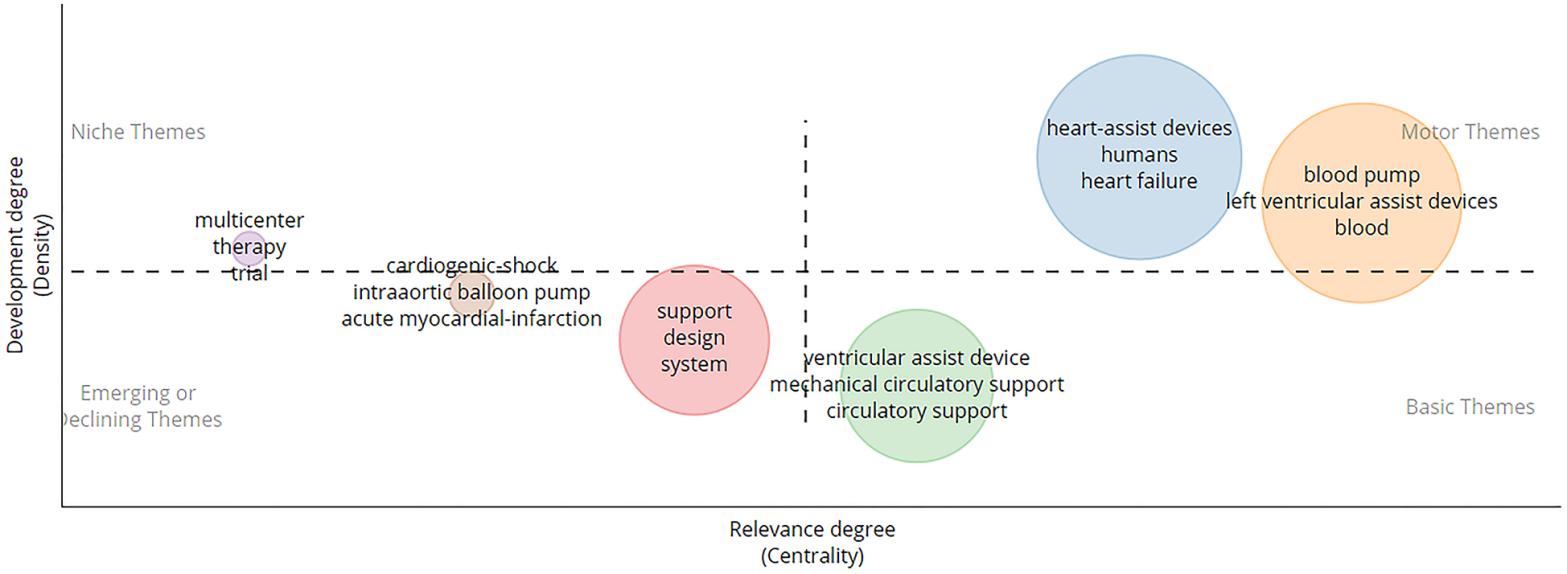
Thematic map showing various clusters related to motor, niche, basic, and emerging themes.

Thematic maps are visualizations of the thematic structure of a research field. They are created by analyzing the keywords in a collection of scholarly documents, and these are grouped into clusters based on their similarity. From Figure 8, we can see four different parts of the thematic map as explained below:

• *Motor themes* are the most important and well-established themes in a research field. From Figure 8, the motor themes include left ventricular assist devices, assisted circulation, heart-assist devices, hemolysis, equipment design, and blood pump.
• *Niche themes* are smaller and less important themes in a research field. Niche themes may be new and emerging topics or may be specialized and focused on a particular aspect of the field. From Figure 8, the niche themes include multicenter therapy and trial. This indicates that a focused and emerging theme in this domain of research is the multicenter trial of VAD-related equipment (61–64).
• *Basic themes* are fundamental themes that are common to many different research fields. From Figure 8, the basic themes include ventricular assist device, pump, flow, and continuous-flow type pump.
• *Emerging or declining themes* are themes that are either new and growing or old and declining. From Figure 8, the emerging terms include multicenter studies (65), wireless (66), machine learning (67), acute myocardial infarction (68), and miniaturization and cardiogenic shock (69).

### 3.10 Emerging trends in the design of VADs: trend topic analysis

**Figure 9:**
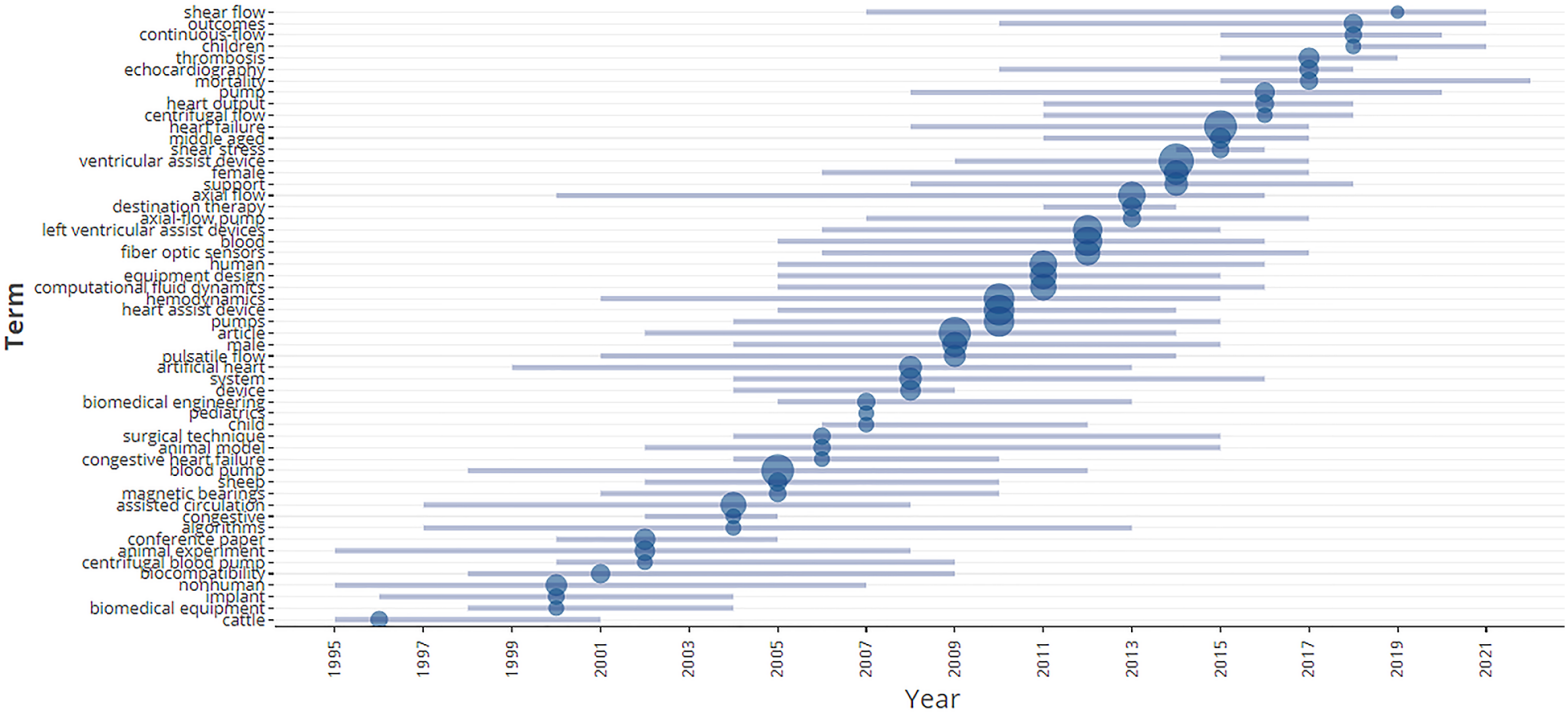
Trend topics year-wise.

Trend topic plots in single-quadrant mode are shown in Figure 9. Single-quadrant trend topic plots show all terms in the same quadrant, with the size of the circles representing the frequency of the terms. The terms with the largest circles represent the most frequently used terms in the field. In a trend topic plot, if a particular term is becoming more common over time, this means that the field is moving in that direction. Figure 9 shows the trend topics in the field of research of VAD design and development, including blood pumps, hemodynamics, fiber-optic sensors, ventricular assist devices, continuous flow, miniaturization, computational fluid dynamics, machine learning, wireless, and shear flow.

### 3.11 Analysis of keyword dynamics

**Figure 10:**
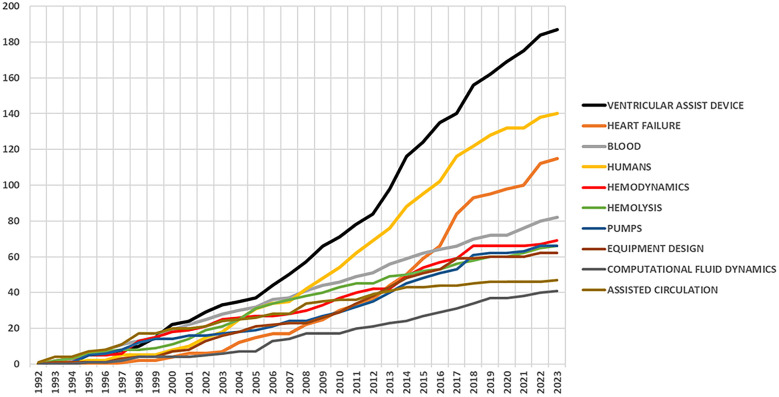
Keyword dynamics of top 10 keywords.

Figure 10 presents the growth of the top 10 keywords with time in this field of study. The keywords “ventricular assist device,” “blood pump,” and “human” are the most noticeable and have been used regularly by the authors over the years. “Hemodynamics,” “computational fluid dynamics,” and “equipment design” are the other significant keywords associated with this study.

### 3.12 Analysis of correlation between citations received and document age

Table 5 highlights the results of the correlation test between the document age (based on publication year) and the number of citations for published papers. The Shapiro–Wilk test of normality showed that a *p*-value less than 0.05 (*p* = 0.000) means distribution was not normal. Using the Spearman rank correlation test, the correlation was tested. A moderate correlation between the two variables was found (*r* = 0.333) suggesting a moderate tendency for older published works to have more citations.

**Table 5:** Correlation between document age and citations.

Correlations				
			Citations	Document age
Spearman's rho	Citations	Correlation coefficient	1.000	0.333**
		Sig. (two-tailed)		0.000
		*N*	489	489
	Document age	Correlation coefficient	0.333**	1.000
		Sig. (two-tailed)	0.000	
		*N*	489	489

** Correlation is significant at the 0.01 level (two-tailed).

## 4 Discussion

To the best of our knowledge, this is the first bibliometric analysis to use merged data from Web of Science and Scopus databases to study the evolution of VAD designs using experimental and computational techniques.

### 4.1 Discussion of results

The modern developments in VAD design and surgery make them a more practicable choice for more patients (70). Our analysis provides insights into the latest trends and advances in this field, which is essential for saving the lives of patients with heart failure or those who need a heart transplant. Many researchers have published papers on VAD design- and development-related topics since the year 1998–2016 (*N* = 327). This is the time zone when the use of second-generation VADs started (71), and research was focused on further improvements in its design which resulted in third-generation VADs starting from 2017 onwards. Since then, researchers have been focusing on improving its design, use, and maintenance through machine learning and artificial intelligence techniques along with some other design improvements.

The top 10 nations leading the charge in this field of research are predominantly developed countries, with Brazil being the notable exception (refer to Table 2). The citation count further underscores the dominance of these developed nations, indicating that the majority of significant research in this domain originates from these countries. This suggests that developing nations have considerable ground to cover in advancing their technology in this field. Among the developing nations, only Brazil, India, Iran, Malaysia, and Thailand have made some noteworthy contributions to the published research in the field related to VAD design and development. Some possible causes for lesser research done by developing countries can be:

• Less availability of resources and funding are some of the biggest causes that prevent researchers from carrying out quality research in this domain. The cost of publishing in reputed publication houses could be another reason as it would be very difficult to fund their publications without any outside support for the researchers.
• The availability of state-of-the-art research infrastructures in the form of specialized labs and skilled manpower is another prominent factor hindering the quality of research work.
• In some developing countries, the priority of the funding by the concerned authorities can be for some other sectors which can be of greater priority as compared to this domain.

From the keyword co-occurrence network (see Figure 6), we see a gradual increase in the number of themes being covered under this domain. More and more relevant areas of concern related to VAD design and development are being explored with time. Some early publications focused on a very preliminary design. As more researchers started working in this domain, we see more new topics being explored related to VAD such as hemodynamics (72), fiber-optic sensors (73), miniaturization (74), machine learning (75), wireless (76), and shear stress (77) as can be also observed from Figure 9. VAD designers are also working to improve designs to reduce blood damage by optimizing the interaction between blood and VAD equipment.

The thematic analysis provided important information regarding the evolution of different topics connected to the technology involved in the design process with new keywords appearing in research such as miniaturization, machine learning, artificial intelligence, and centrifugal pumps. The themes appearing in four groups of thematic maps (motor, niche, basic, and emerging) help us understand that few themes are isolated and focus on a particular area of research only, such as multicenter trials, and also help us identify trending topics in this research domain, such as machine learning, artificial intelligence, fiber-optic sensors, and continuous-flow pump.

One of the most important areas that are of particular interest in the ongoing research related to improvement in the design of LVADs is trials of LVAD in multicenters (78). These multicenter studies are of great significance as these studies provide data from multiple clinics and hospitals which need not necessarily be in one region, and this type of study can be done across multiple countries also. This helps the researchers in getting a bigger data set and that also from diverse backgrounds of patients of different age groups and diverse medical backgrounds. With this, multicenter studies have a greater chance of providing a more realistic set of data for researchers to analyze and design new LVADs based on this information. A large number of researchers are already working on using data collected from multicenter studies across the globe (79–81).

### 4.2 Significance

This analysis can be used by different groups of researchers to understand the social and practical implications of the topic.

A. Social implications

Public awareness can be increased as people get to know about the various advancements in the design process of LVADs taking place across the globe. Readers will also get some insight into the historical evolution of the design of these devices with time and how researchers are trying to improve the quality of life of patients using these devices.

B. Practical implications

Key areas that need improvement while designing LVAD can be identified. The biocompatibility and durability of these devices can also be improved by following the latest trend topics in this research domain. The use of emerging techniques such as machine learning and artificial intelligence can be helpful for future researchers. This bibliometric analysis can used to find focused research areas that need more funding and grants. The information obtained from analysis such as top-cited works, top countries, and authors and their collaboration can help researchers study, compare, and analyze the existing techniques and find ways to improve them further.

### 4.3 Limitations and future directions

There are a few limitations of this work since only Biblioshiny software by RStudio and Microsoft Excel have been used in conducting bibliometric analysis. However, there are various other bibliometric tools available such as CiteSpace and VOSviewer. Another limitation is the possibility of having missed out on some of the published papers related to this domain of research during the data collection process. Papers written in the English language alone have been included in this work. Another limitation is that bibliometric analysis uses only published papers so unpublished work is not included in this study. Moreover, as only two sources (Scopus and WoS) were used for data collection, some published works that are not part of these databases were also not included in this study.

The areas in which further work can be done to improve the existing designs of the LVADs are discussed as follows:

• Minimising damage to blood cells: LVADs which are presently available cannot guarantee zero damage to blood cells because of the interaction of the blood with the device and due to shearing action. This can lead to some complications such as bleeding or clotting in some patients. Researchers are working to address this problem by decreasing damage to blood cells during the interaction of blood with these devices, and this field is still open to future research.
• Reduction in device size and weight: Research is ongoing to optimize the size and weight and also improve the efficiency of the currently available LVADs to make them more comfortable and safer for the patients.
• On-demand design: Researchers can focus on designs based on the needs of patients as the same size and design might not be optimal for all patients, and patient-specific manufacturing (by using 3D printing techniques and sensor-based controls) can be an option to improve patient comfort and LVAD performance.
• Use of less invasive surgical procedures: To improve patient comfort and reduce the post-implantation LVAD recovery period, the researchers are focussing on finding ways to achieve this and improve such existing techniques.
• One promising area of research is wireless VADs, making them more comfortable and reducing the risk of infection.
• Using patient's blood as a power source: Another promising area of research is implantable VADs powered by the patient's blood flow, eliminating the need for a heavy battery and improving patient activity levels.
• Improving destination therapy (DT) VADs: In the case of destination therapy VADs, the costs involved can be high, and further research is needed in this area to make it more affordable and also to improve the functionality of these.
• Use of machine learning and artificial intelligence: The use of machine learning and artificial intelligence can be made to help improve the existing designs and also to make novel designs based on information available from existing designs and the requirements of patients.
• Research beyond LVADS: Although LVADs have been widely used devices, researchers have started to look beyond LVADS. Biventricular assist devices (BiVADs) and total artificial hearts (TAH) are attracting more researchers, and there is a large scope in these domains.

## 5 Conclusions

This paper presents the first-ever bibliometric analysis of the design and development of VADs using merged data from the Scopus and WoS databases. The study provides a comprehensive overview of the research in this domain, including the most influential authors, publications, network analysis, thematic evolution, and trend topics. This study provides valuable insights into the field and highlights the need for continuous exploration to keep up with rapidly changing technologies. The study also identifies several emerging themes in this digital age, such as miniaturization, artificial intelligence, machine learning, wireless, and shear flow, which warrant further investigation to better understand the new challenges in this field. Ventricular assist device (VAD) technology is rapidly advancing, with new devices being developed all the time. Ventricular assist devices (VADs) have radically enhanced the quality of life for patients with heart-related issues needing treatment. VADs can support patients in living longer and more active lives.

## Data Availability

Publicly available datasets were analyzed in this study and are included in the article/supplementary material. This data can be found here: www.scopus.com and www.webofscience.com and further inquiries can be directed to the corresponding author.
